# Psychosocial job conditions and biomarkers of cardiovascular disease: A cross-sectional study in the Swedish CArdioPulmonary bioImage Study (SCAPIS)

**DOI:** 10.1177/14034948211064097

**Published:** 2022-01-06

**Authors:** Mia Söderberg, Helena Eriksson, Kjell Torén, Göran Bergström, Eva Andersson, Annika Rosengren

**Affiliations:** 1 Occupational and Environmental Medicine, School of Public Health and Community Medicine, University of Gothenburg, Sweden; 2 Department of Occupational and Environmental Medicine, Sahlgrenska University Hospital, Sweden; 3 Department of Molecular and Clinical Medicine, Institute of Medicine, University of Gothenburg, Sweden

**Keywords:** Job demand–control, hypertension, cholesterol, coronary artery calcification, metabolic syndrome, cardiovascular disease

## Abstract

**Aims:**

The aim of this study was to investigate associations between psychosocial work exposure and the presence of biological and imaging biomarkers of cardiovascular disease.

**Methods:**

This cross-sectional study was conducted in a sub-cohort of the Swedish CArdioPulmonary bioImage Study (SCAPIS). Psychosocial exposure was evaluated with the job demand–control model, and analysed according to the standard categorization: high strain, active, passive and low strain (reference). Biomarkers (blood pressure, high-density lipoprotein (HDL) and low-density lipoprotein (LDL) cholesterol, coronary artery calcification (CAC) and metabolic syndrome) were measured, or derived through measurements, from clinical examinations. Gender-specific prevalence ratios (PRs) and 95% confidence intervals (CIs) were calculated with regression models and adjusted for age, education, smoking, physical activity, general life stress and body mass index (BMI).

**Results:**

The analyses included 3882 participants (52.5% women). High strain (high demands–low control) was linked to increased PR for low HDL cholesterol in women, adjusted for all covariates (PR 1.76; 95% CI 1.25–2.48). High strain was also related to moderately increased PR for metabolic syndrome in men, after adjustments for all covariates except BMI (PR 1.25; 95% CI 1.02–1.52). In addition, passive work (low demands–low control) was associated with diastolic hypertension in women (fully adjusted: PR 1.29; 95% CI 1.05–1.59). All relationships between psychosocial factors and LDL cholesterol or CAC (both genders), or hypertension (men), were non-significant.

**Conclusions:**

**Poor psychosocial job conditions was associated with the presence of low HDL cholesterol and diastolic hypertension in women, and metabolic syndrome in men. These findings contribute to the knowledge of potential pathways between stressful work and coronary heart disease.**

## Introduction

Widespread evidence relates adverse psychosocial work conditions to coronary heart disease (CHD). The most influential psychosocial model is the job demand–control (JDC) model [1–3], which operationalizes a combination of job demand and buffering effects from job control. Although experimental studies are lacking, several longitudinal studies have linked high demands–low control, referred to as high strain, to CHD [2,4]. A recent meta-analysis, based on 46 pooled prospective cohort studies, showed a 34% increased hazard ratio (95% confidence interval (CI): 18–51%) for CHD in high-strained workers [5]. Hazard ratios were higher in women than men [5], but similarities in health risks across different age groups and countries have been observed in other studies [4]. Still, the role of intermediary pathways, such as the presence of biomarkers of cardiovascular disease – for example, hypertension, high cholesterol levels or obesity – remain unclear [6].

Hypertension is one of the most investigated outcomes in this context, but results are inconsistent [7,8] and conclusions on high strain as a risk factor for clinical hypertension cannot be drawn. One paper, conducting both a review study and meta-analysis, focusing on ambulatory blood pressure, found an influence of work stressors on blood pressure, as ambulatory systolic blood pressure (SBP) was lower during days off than during workdays [8]. However, the meta-analysis in the same paper noted that, even though blood pressure appeared to be raised during periods of high strain, this exposure was not related to increased 24-hour ambulatory blood pressure. Since most studies are based on small samples or do not stratify by gender, the role of stressing exposure as a causal factor in hypertension may still be under-investigated.

Other biomarkers – for example, cholesterol – are less studied, and some findings even illustrate lower total cholesterol in men and women aged 15–64 with high-strain work [9], but a relatively large proportion of younger persons in these studies may have obscured associations. Yet another study found no associations for high strain, but instead found that a passive work environment (low demands–low control) was associated with elevated total cholesterol and low-density lipoprotein (LDL) cholesterol [10].

In the light of conflicting results, coronary atherosclerosis, a well-known biomarker for increased risk of future clinical CHD, is gaining attention. The majority of studies examining psychosocial job aspects and atherosclerosis have used carotid intima media thickness (IMT), which carries the benefit of data collection through non-invasive methods. But results have been inconclusive [11–13] and IMT does not reflect a direct representation of the coronary vasculature. The presence of coronary artery calcification (CAC) is a marker of atherosclerosis in the coronary arteries and a predictor for CHD. The more refined method of measuring CAC, through visualizing calcium in the coronary arteries, could provide better insight in the build-up towards clinical CHD. A higher prevalence of CAC has been observed in persons with chronic life stress [7] and depression [14], which triggers similar physiobiological mechanisms as external stressors, but little is known about relations to job exposure. In the longitudinal Coronary Artery Risk Development in Young Adults study, no associations between CAC and job strain was found, potentially due to the young age of the participants, 38–50 years at follow-up [15], or that JDC was recorded up to 20 years before evaluating CAC. Similarly, in a cross-sectional study of 1849 persons, no associations with job strain were found [16].

It is also likely that no single variable is predictive for CHD and, therefore, some focus has shifted towards metabolic syndrome, defined as the presence of a cluster of CHD risk factors – that is, hypertension, waist circumference, elevated fasting glucose and blood lipids. Few studies have examined relationships between psychosocial job conditions and metabolic syndrome, but both high strain and shift work may potentially be linked to the presence of metabolic syndrome [17].

This study aims to investigate associations between adverse psychosocial work conditions and biomarkers of cardiovascular heart disease, based on the hypothesis that stressful psychosocial work exposure, principally high strain, increases the prevalence of these biomarkers.

## Methods

### Procedure and participants

Subjects analysed in this study constituted a sub-cohort of the large population-based cohort, SCAPIS (Swedish CArdioPulmonary bioImage Study) [18]. The full cohort consists of randomly selected men and women (*n* = 30,154, participation rate 49.5%) from the general Swedish population, aged 50–64 years. Data collection was carried out at six sites in Sweden, between November 2013 and November 2018, according to an extensive clinical examination protocol, lasting over 2–3 days within a two-week period, and including questionnaires capturing medical history, lifestyle and personality traits.

**Figure 1.**
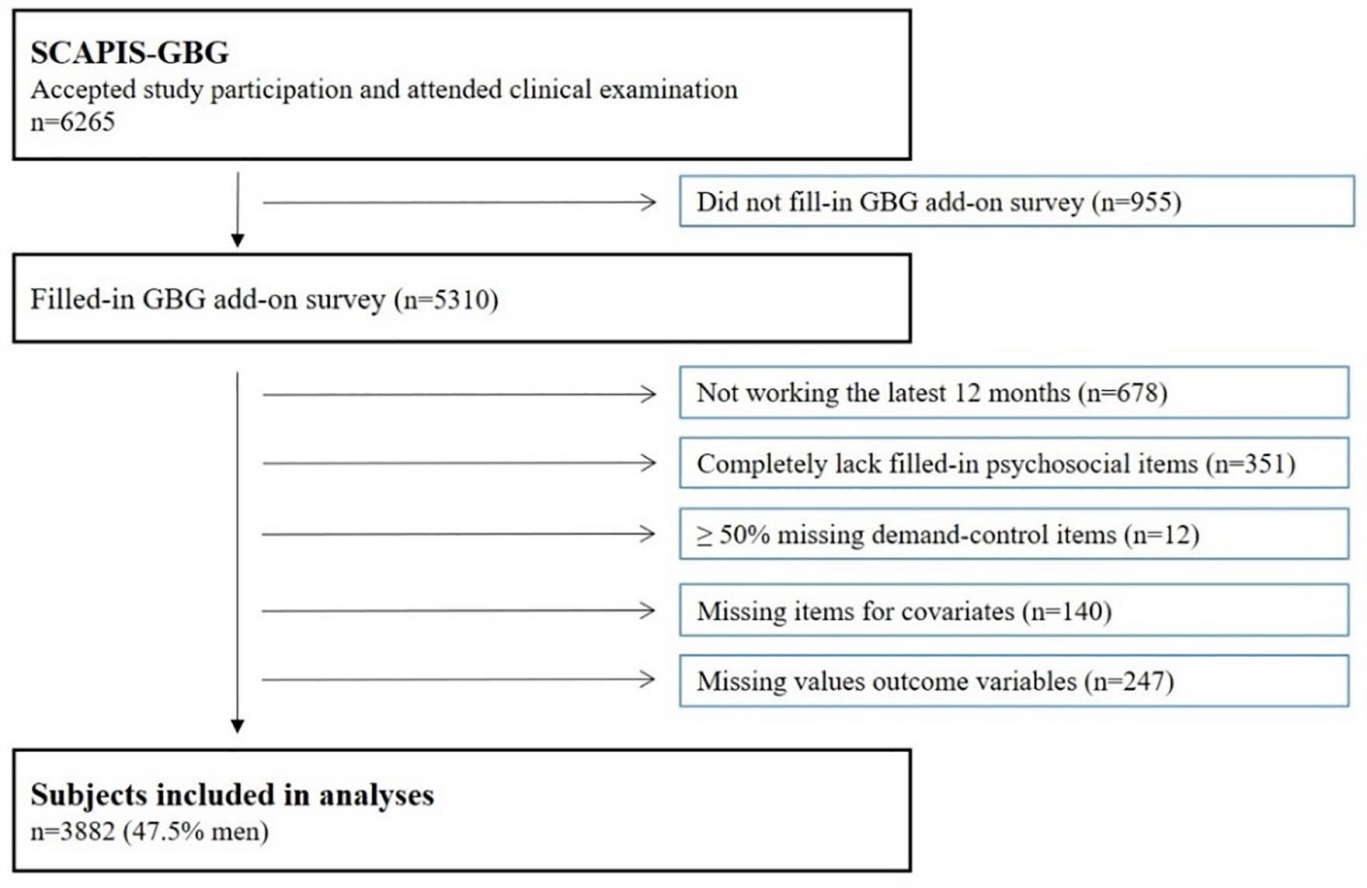
Flowchart of studied subjects, Gothenburg (GBG).

The current study is based on participants included at the Gothenburg site (*n* = 6265). Each regional site collected additional data to enable local research interests. At the Gothenburg site, participants were asked to fill in an additional questionnaire focusing on work environment, which was completed by 5310 (85%) of the subjects. The lower response rate relates to that many participants brought this questionnaire home, but never returned it. For the analyses, the following exclusions were made: not working the last 12 months (*n* = 678), completely lacking filled-in psychosocial items (*n* = 351), missing ⩾50% of filled-in job demand or control items (*n* = 12), lacking filled-in items for the selected covariates (*n* = 140) or lacking valid values for the biomarkers (*n* = 247). A flowchart of the participants is presented in Figure 1.

All procedures were conducted in accordance with the ethical standards of the 1964 Helsinki Declaration. Ethical approval for the study was granted by the Ethical Review Board of Gothenburg (896-18) and the Ethical Review Board of Umeå, Sweden (2010-228-31M). The participants provided written informed consent.

### Measures

#### Demand–control variables

JDC was measured with the Swedish demand–control–support questionnaire [19]. Job demand and job control were measured with five and six items, respectively. Each item was scored on a four-point scale (1–4), ranging from “Often” to “Almost never”. A sample demand item was, “Does your job require you to work very fast?”, and an example of a control item was, “Do you have the possibility to decide for yourself how to carry out your work?”. By standard procedure, all items were positively inverted so that a high score equated either high demands or high control, and then added up separately (Σ_demand_ / Σ_control)_. Since sum scores were used, missing values needed to be addressed. Subjects lacking ⩾50% filled-in items for either demand or control were excluded (*n* = 12). Subjects with missing items, but less than 50% missing items for either variable, received imputed values, consisting of the mean scores of the remaining items in each variable on an individual level (subjects with imputed scores: demand *n* = 8, control *n* = 6). The sum scores of job demand or control were then dichotomized into high/low by the median values of the distributions, and combined into *high strain* (high demand–low control), *active* (high demand–high control), *passive* (low demand–low control) and *low strain* (low demand–high control). In the regression analyses, low strain was used as reference.

#### Biomarkers

Diastolic blood pressure (DBP) and SBP were both measured twice, in the supine position, after five minutes’ rest, using an automatic device (Omron M10-IT, Omron Healthcare Co., Kyoto, Japan) with the means of each of the two measurements used for analysis. DBP ⩾85 mmHg or SBP ⩾130 mmHg were defined as diastolic or systolic hypertension, or, for either, the use of antihypertensive medication. High-density lipoprotein (HDL) cholesterol, LDL cholesterol and plasma glucose were analysed through standard analyses at an accredited laboratory. Low HDL cholesterol was defined as <1.03 mmol/L in men and <1.30 mmol/L in women. High LDL cholesterol was defined as ⩾3.5 mmol/L in men and ⩾4.2 mmol/L in women. For both HDL and LDL cholesterol, subjects were also considered as cases if reporting treatment with statin medication.

CAC was measured by using a multi-slice computed tomography scanner (Siemens Acuson S2000 ultrasound scanner equipped with a 9L4 linear transducer, both from Siemens, Forchheim, Germany) [18]. The two-dimensional greyscale ultrasound image was analysed to determine plaque size and number of plaques. The calcium content in each coronary artery was measured and summed to produce a total coronary artery calciﬁcation score (CACS) according to international standards [20], using the Agatston score [21]. CAC was defined as a CAC-score (CACS) >0, which represents the presence of detectable calcification.

Metabolic syndrome was a derived variable according to criteria by the American Heart Association [22]. The participants were classified as having metabolic syndrome or not, based on the presence of any three out of five parameters. The five parameters were as follows: elevated waist circumference (⩾88 cm in women; ⩾102 cm in men), elevated triglycerides (⩾1.7 mmol/l or drug treatment for elevated triglycerides), reduced HDL cholesterol (<1.3 mmol/l in women; <1.03 mmol/l in men) or treatment with statins, elevated blood pressure (SBP ⩾130 or DBP ⩾85) or hypertensive drug treatment, elevated fasting glucose (⩾5.5 mmol/l) or treatment with antidiabetic drugs or insulin.

#### Covariates

Several covariates were considered: age, education, smoking, physical exercise, general life stress and BMI. Age was entered as a continuous variable. Smoking was analysed as a categorical variable: current smoker, ex-smoker, never-smoker (reference). Education was defined as the highest education finished. Physical exercise was measured using one item, “How often have you exercised or worked out in exercise clothes during the last three months to improve your fitness and/or to feel good?”. Response options (1–4) ranged from 1 = “Never” to 4 = “More than three times a week” [23]. Life stress was captured with the item, “By stress we mean feeling tense, irritable, anxious or having sleeping difficulties as a result of conditions at work or at home. Did you experience this?”, with responses (0–4) ranging from 0 = “Never experienced this” to 4 = “Constant stress the latest five years” [24]. The variables for education, physical exercise and general life stress were dichotomized into higher education (yes/no), weekly exercise (yes/no) and constant life stress during the latest 1–5 years (yes/no). BMI was calculated by weight in kilograms, divided by height in squared meters.

#### Statistical analysis

Statistical analyses were performed with statistical software SAS (version 9.4 for Windows, SAS Institute, Cary, NC). Prevalence ratios (PRs) and 95% CIs between psychosocial exposure and biomarker variables were calculated with Poisson regression with robust variance and log link. Poisson regression is primarily used in survival analyses, but when cases constitute >10%, methods such as logistic regression tend to overestimate the strength of the relationships, while Poisson regression has been found to provide more correct estimates [25]. As the proportion of cases for most biomarkers constituted more than 10% of the participants, this method was chosen. A 95% CI not including 1 was considered as statistically significant. The chosen covariates were entered stepwise into three models. The first model only included age as a covariate. The second model included age, education, smoking, physical exercise and general life stress. In the third model, BMI was added to the same covariates as in model 2. All association to biomarkers were analysed with these three models, except metabolic syndrome, which was only analysed with model 1 and 2, since waist circumference, used to derive the variable for metabolic syndrome, reflect similar aspects as BMI. Each biomarker was analysed in a separate model, stratified by gender.

## Results

In total 3882 participants were included in our analyses (52.5% women). The characteristics of the participants according to JDC category and gender are shown in Table I. About 10% of the men and 18% of the women reported high-strain work conditions. Higher education and weekly exercise were more common in active workers, while persons with high strain more often reported smoking. Both high-strained and active workers more often experienced general life stress.

**Table I.** Cohort characteristics by job demand–control categories and gender.

Variable	Men								Women							
	High strain		Active		Passive		Low strain		High strain		Active		Passive		Low strain	
*n*	186	(10.1)	423	(23.0)	416	(22.6)	818	(44.4)	362	(17.8)	496	(24.3)	483	(23.7)	698	(34.2)
Age, mean (SD)	56.9	(4.1)	56.8	(4.1)	57.5	(4.2)	57.5	(4.3)	56.8	(3.9)	57.0	(4.1)	57.1	(4.2)	57.4	(4.3)
Higher education (=yes), *N* (%)	60	(32.4)	238	(56.3)	120	(28.9)	372	(45.5)	143	(39.6)	359	(72.4)	171	(35.5)	416	(59.8)
Current smokers, *N* (%)	36	(19.4)	49	(11.6)	46	(11.1)	95	(11.6)	54	(14.9)	73	(14.7)	52	(10.8)	90	(12.9)
Never-smoker, *N* (%)	90	(48.4)	244	(57.7)	214	(51.4)	440	(53.8)	176	(48.6)	225	(45.4)	214	(44.3)	316	(45.3)
General life stress the latest 1–5 years (=yes), *N* (%)	61	(32.8)	125	(29.6)	35	(8.4)	67	(8.2)	160	(44.2)	197	(39.7)	72	(14.9)	103	(14.8)
Weekly exercise (=yes), *N* (%)	66	(35.5)	231	(54.6)	210	(50.5)	456	(55.8)	163	(45.0)	276	(55.7)	251	(52.0)	389	(55.7)
BMI (kg/m^2^), mean (SD)	27.6	(3.9)	26.9	(3.8)	27.2	(3.8)	26.9	(3.5)	26.1	(4.2)	26.0	(4.4)	26.3	(4.7)	25.5	(4.4)
Waist circumference (cm), mean (SD)	99.3	(11.0)	97.6	(10.8)	99.2	(11.4)	98.2	(10.0)	88.3	(11.5)	87.7	(11.7)	87.9	(12.0)	86.6	(11.7)
Triglycerides (mmol/L), mean (SD)	0.3	(0.5)	0.2	(0.4)	0.3	(0.4)	0.2	(0.4)	0.1	(0.3)	0.1	(0.3)	0.1	(0.3)	0.1	(0.3)
Plasma glucose (mmol/L), mean (SD)	6.1	(1.7)	5.9	(1.0)	5.9	(0.9)	5.8	(0.9)	5.6	(1.2)	5.4	(0.9)	5.5	(1.0)	5.4	(0.7)
DBP, mean (SD) (mmHg)	74.5	(9.5)	74.3	(10.2)	75.3	(10.1)	75.2	(9.9)	72.0	(10.8)	70.7	(10.3)	72.7	(10.7)	70.8	(10.0)
Hypertension DBP, *N* (%)^a^	58	(31.2)	119	(28.1)	134	(32.2)	234	(28.6)	91	(25.1)	98	(19.8)	131	(27.1)	135	(19.3)
SBP, mean (SD) (mmHg)	126.3	(14.7)	126.1	(15.5)	126.6	(14.5)	127.0	(15.0)	117.4	(17.3)	117.1	(17.0)	119.9	(17.0)	117.5	(17.2)
Hypertension SBP, *N* (%)^b^	95	(51.1)	171	(40.4)	198	(47.6)	370	(45.2)	116	(32.0)	147	(29.6)	166	(34.4)	192	(27.5)
HDL, mean (SD) (mmol/L)	1.4	(0.4)	1.5	(0.4)	1.5	(0.4)	1.5	(0.4)	1.9	(0.5)	1.9	(0.5)	1.9	(0.5)	2.0	(0.5)
Low HDL, *N* (%)^c^	51	(27.4)	82	(19.4)	91	(21.9)	153	(18.7)	64	(17.7)	58	(11.7)	58	(12.0)	66	(9.5)
LDL, mean (SD) (mmol/L)	3.6	(0.9)	3.7	(0.9)	3.6	(0.9)	3.7	(0.9)	3.6	(0.9)	3.7	(0.9)	3.7	(0.9)	3.7	(1.0)
High LDL, *N* (%)^d^	65	(35.0)	160	(37.8)	147	(35.3)	316	(38.6)	125	(34.5)	164	(33.1)	162	(33.5)	241	(34.5)
CACS, mean (SD)	58.0	(146.5)	62.7	(181.1)	103.9	(360.2)	85.4	(254.2)	18.1	(78.0)	24.4	(109.9)	21.9	(158.1)	18.3	(76.5)
CACS >0, *N* (%)	103	(55.4)	219	(51.8)	226	(54.3)	434	(53.1)	73	(20.2)	129	(26.0)	124	(25.7)	168	(24.1)
Metabolic syndrome, *N* (%)^e^	81	(43.6)	133	(31.4)	150	(36.1)	248	(30.3)	90	(24.9)	94	(19.0)	103	(21.3)	125	(17.9)

SD: standard deviation; BMI: body mass index; DBP: diastolic blood pressure; SBP: systolic blood pressure; HDL: high-density lipoprotein; LDL: low-density lipoprotein; CACS: coronary artery calciﬁcation score.

a DBP ⩾85 mmHg or use of hypertension medication.

b SBP⩾130 mmHg or use of hypertension medication.

c HDL cholesterol (men) <1.03 mmol/L or (women) <1.30 mmol/L or use of statin medication.

d LDL cholesterol (men) ⩾3.5mmol/L or (women) ⩾4.2 mmol/L or use of statin medication.

e The presence of three out of five parameters: waist circumference (⩾102 cm in men; ⩾88 cm in women), triglycerides (⩾1.7 mmol/l or use of statin medication), HDL cholesterol (<1.03 mmol/l in men; <1.3 mmol/l in women; or use of statin medication), blood pressure (SBP ⩾130 or DBP ⩾85; or use of hypertension medication), fasting glucose (⩾5.5 mmol/l or treatment with antidiabetic drugs or insulin).

Poisson regression analyses illustrated pronounced gender differences with respect to associations between psychosocial exposure and the examined biomarkers (Table II). In men, all analyses between psychosocial factors and hypertension displayed small and non-significant effects. For women, both high strain and passive work were associated with diastolic and systolic hypertension (model 1), compared to women with low strain. Effects for high strain decreased to non-significance in the fully adjusted models, but persisted in women with a passive work environment (DBP: PR 1.29; 95% CI 1.05–1.59).

**Table II.** Poisson regression for associations between job demand–control and biomarkers for coronary heart disease.

	Hypertension DBP			Hypertension SBP			Low HDL-cholesterol			High LDL-cholesterol			Coronary artery calcification			Metabolic syndrome		
	*Cases*	*PR*	*95% CI*	*Cases*	*PR*	*95% CI*	*Cases*	*PR*	*95% CI*	*Cases*	*PR*	*95% CI*	*Cases*	*PR*	*95% CI*	*Cases*	*PR*	*95% CI*
**Men**																		
High strain^a^	58	1.13	0.89–1.43	95	1.16	0.99–1.36	51	**1.48**	**1.12–1.94**	65	0.91	0.73–1.13	103	1.08	0.94–1.24	81	**1.46**	**1.20–1.77**
Active^a^	119	1.03	0.85–1.23	171	0.92	0.81–1.06	82	1.04	0.82–1.32	160	0.99	0.85–1.15	219	1.01	0.90–1.13	133	1.06	0.89–1.27
Passive^a^	134	1.14	0.96–1.36	198	1.06	0.94–1.20	91	1.17	0.93–1.48	147	0.92	0.78–1.07	226	1.04	0.93–1.15	150	**1.20**	**1.01–1.41**
High strain^b^	58	0.97	0.76–1.25	95	1.11	0.94–1.31	51	1.33	0.99–1.77	65	0.91	0.73–1.15	103	1.08	0.93–1.26	81	**1.25**	**1.02–1.52**
Active^b^	119	1.03	0.85–1.25	171	0.95	0.82–1.09	82	0.99	0.77–1.26	160	0.97	0.83–1.14	219	1.01	0.90–1.13	133	1.04	0.87–1.25
Passive^b^	134	1.10	0.92–1.31	198	1.03	0.91–1.16	91	1.13	0.89–1.42	147	0.91	0.78–1.07	226	1.04	0.93–1.15	150	1.14	0.97–1.34
High strain^c^	58	0.94	0.73–1.20	95	1.08	0.92–1.26	51	1.28	0.95–1.72	65	0.91	0.72–1.14	103	1.07	0.92–1.24		N/A	
Active^c^	119	1.00	0.83–1.21	171	0.93	0.81–1.07	82	0.95	0.74–1.22	160	0.97	0.83–1.13	219	1.01	0.90–1.13		N/A	
Passive^c^	134	1.09	0.91–1.29	198	1.02	0.90–1.15	91	1.11	0.88–1.40	147	0.91	0.78–1.06	226	1.04	0.93–1.15		N/A	
**Women**																		
High strain^a^	91	**1.35**	**1.07–1.70**	116	**1.21**	**1.00–1.47**	64	**1.90**	**1.38–2.61**	125	1.05	0.88–1.25	73	0.91	0.72–1.16	90	**1.45**	**1.15–1.85**
Active^a^	98	1.04	0.82–1.31	147	1.10	0.92–1.31	58	1.26	0.91–1.76	164	0.98	0.84–1.15	129	1.13	0.93–1.36	94	1.09	0.86–1.38
Passive^a^	131	**1.42**	**1.15–1.76**	166	**1.28**	**1.08–1.51**	58	1.29	0.92–1.79	162	0.99	0.84–1.16	124	1.10	0.90–1.33	103	1.22	0.97–1.53
High strain^b^	91	1.20	0.93–1.55	116	1.08	0.88–1.32	64	**1.71**	**1.20–2.45**	125	0.99	0.83–1.19	73	0.81	0.62–1.05	90	1.18	0.92–1.52
Active^b^	98	1.01	0.79–1.29	147	1.09	0.90–1.32	58	1.26	0.88–1.81	164	0.98	0.83–1.16	129	1.15	0.94–1.41	94	1.04	0.87–1.25
Passive^b^	131	**1.37**	**1.10–1.70**	166	**1.21**	**1.01–1.44**	58	1.18	0.85–1.65	162	0.94	0.80–1.11	124	1.04	0.85–1.28	103	1.09	0.86–1.37
High strain^c^	91	1.23	0.96–1.57	116	1.09	0.90–1.33	64	**1.76**	**1.25–2.48**	125	0.99	0.83–1.19	73	0.81	0.62–1.05		N/A	
Active^c^	98	0.97	0.76–1.23	147	1.06	0.88–1.28	58	1.21	0.85–1.71	164	0.97	0.82–1.15	129	1.13	0.92–1.39		N/A	
Passive^c^	131	**1.29**	**1.05–1.59**	166	1.15	0.97–1.36	58	1.12	0.81–1.54	162	0.92	0.78–1.09	124	1.03	0.83–1.26		N/A	

PR: prevalence ratio; CI: confidence interval; DBP: diastolic blood pressure; SBP: systolic blood pressure; HDL: high-density lipoprotein; LDL: low-density lipoprotein. Reference: low strain.

a Model 1: adjusted for age.

b Model 2: model 1 + higher education, smoking, physical activity, general life stress.

c Model 3: model 2 + body mass index.

Bold indicate statistically significant

High strain was linked to low HDL cholesterol in both men (PR 1.48; 95% CI 1.12–1.94) and women (PR 1.90; 95% CI 1.38–2.61) in age-adjusted models. These findings remained significant in women, but turned borderline non-significant in men when fully adjusted, illustrating a 76% (95% CI 25–148%) increased PR for low HDL cholesterol. In contrast, no significant associations between psychosocial variables and LDL cholesterol or CAC were found. High strain was associated with metabolic syndrome in both men and women, but only remained statistically significant in men when entering the covariates for model 2 (PR 1.25; 95% CI 1.02–1.52). Analyses between single variables (job demand or job control) and cardiovascular disease biomarkers were also carried out. However, these analyses mostly displayed small effects and non-significant associations in the fully adjusted model (Appendix 1), except for relationships between job control and DBP hypertonia in women (PR 0.84; 95% CI 0.72–0.98).

## Discussion

High strain, regarded as the most hazardous psychosocial job exposure, was related to notably increased PR for low HDL cholesterol in women and moderately augmented PR for metabolic syndrome in men, while passive work was associated with diastolic hypertension in women. Most associations between job strain variables and hypertension or cholesterol were non-significant among men. Similarly, all associations between JDC and CAC were non-significant in both men and women.

Considering the vast amount of literature identifying stressful psychosocial work environment as a risk factor for CHD [2,4], it is urgent to establish the intermediary biological pathways, through which external straining exposure leads up to CHD events. Such knowledge may improve efficiency in both workplace interventions and medical treatment. Although much research has aimed to expose such chains mechanisms, by analysing outcomes measured with new techniques, we had hoped to contribute to such knowledge.

Our partly contradictory results of associations between high strain and hypertension are similar to previous findings [7,8]. One meta-analysis of pooled cross-sectional data found no effects of high strain on either DBP or SBP [26], but the study used a different reference value – that is, all types of JDC exposure except high strain, instead of using low strain (low demands–high control). Another study, based on participants drawn from the same source population as this study (the general population of Gothenburg), only found increased prevalence for hypertension in men with high strain [10], while this study only found increased prevalence of diastolic hypertension in high-strained women. Some argue that the inconsistent results relate to the fact that blood pressure is often measured at one time-point only, and in surroundings unfamiliar to the participant, but one meta-analysis, investigating ambulatory blood pressure over 24 hours, also failed to find associations with high strain [8].

The most conspicuous result in our study, a 76% increased PR (95% CI 25–148%) for low HDL cholesterol, was found among women with high strain. Increased PR was also found in high-strained men, but associations became non-significant in the fully adjusted models. Blood lipids are, in this context, less studied, and links to psychosocial exposure have been difficult to prove. One recent meta-analysis with pooled data from a cross-sectional study including 47,000 men and women could not establish any associations between job strain and HDL cholesterol. A handful of studies based on the general population from Gothenburg, but with older data from the 1990s [9,10,27], also lacked significant findings for high strain, while passive work was associated with increased total cholesterol and LDL cholesterol in men aged 24–71 years [10]. Two other studies only found small and non-significant effects on total serum cholesterol [27] or even lower odds ratios for total cholesterol in men and women with high strain [9].

A novel part of our study was the inclusion of CAC, measured with a multi-slice computed tomography scanner. However, similar to existing studies, effects were mostly small and non-significant for both genders [15,16]. As development of CAC typically occurs in older adults, lack of findings may relate to the age of the participants (50–65 years). Although, a newly published study based on the full SCAPIS cohort (*n* = 30,154) illustrated the presence of coronary atherosclerosis, detected with coronary computed tomography angiography, in 9.2% of the participants with a CAC-score of 0 [28]. Thus, it is plausible that some participants in our study has developed calcification in vascular beds undetected by CAC. Furthermore, our study design is cross-sectional, but CAC is a slowly progressing disease. One review study, examining psychosocial work exposure, stress appraisal and cardiovascular diseases, concluded that few studies can establish whether longer duration of stress exposure is required to develop slowly progressing diseases [29]. Thus, methods to assess the impact of cumulative psychosocial exposure might be essential to evaluate risks for CAC.

In the present study, the less studied psychosocial work condition, passive work (low demand–low control), was associated with an increased PR for hypertension. A few studies have shown that passive jobs could be associated with myocardial infarction [30] or increased total cholesterol and LDL cholesterol [10]. Due to the sparse literature, we can only speculate, but an underlying reason may be that being under-stimulated also serves as a type of stressor. Another interpretation could be that a passive work situation is linked to an adverse lifestyle, as this work type has been associated with less physical activity during leisure time [31].

There were notable gender differences in the association between JDC variables and biomarkers for CHD. Many of the earlier studies did not stratify by gender, which is unfortunate considering differences in biological risks and the gender-segregated labour market. Findings in existing gender-stratified studies have mostly found relationships between high strain and impaired health in men, and weak or no relationships in women [32]. It has been argued that lack of findings for poor work conditions relates to a more multi-factorial stress exposure for women – that is, a combination of both work and off-work stressors [33]. It may also relate to that women develop cardiovascular disease later in life than men, with disease onset after leaving working life, making our findings foremost in women even more conspicuous. A more recent study based on 13 pooled studies found higher hazard ratios for women with high strain (HR 1.46; 95% CI 1.07–1.99) than for men (HR 1.29; 95% CI) [4], which could indicate a shift in psychosocial work exposures for women or less impact from off-work stressors.

As our study, similar to the existing literature, displays conflicting results in associations between psychosocial exposures and biomarkers for cardiovascular disease, it is plausible that the main pathway is determined by other, possible unknown factors, or that adverse exposure in itself is not the main cause. It has been suggested that high strain has the most damaging effect in the presence of pre-existing vulnerabilities or in persons in low socioeconomic groups, who lack resources to balance the health hazards of stressful work. Future similar studies would benefit from a longitudinal design to establish causality, but also by investigate joint effects from both psychosocial exposure and markers of socioeconomic status.

## Strengths

This study uses a large cohort, with outcomes measured by trained healthcare staff, and CAC measured with new technology. Our analyses make use of all of the JDC model categories, unlike many other studies that set the reference value as a combination of all work types that are not high strain [4]. Such methods may diminish effects since previously healthy work environments – for example, active work or overlooked adverse effects from passive work – may constitute health risks in the modern complex labour market. Many previous studies have not found adverse effects of work conditions, potentially because of study participants that were mostly of an age where the prevalence of risk factors or clinical disease was still low, and, therefore, it could be considered an advantage that our study subjects constitute the “older” age span (50–64 years) of the working population.

## Limitations

The main limitation of this study is the cross-sectional design, which precludes any conclusion of causality, although several longitudinal studies have illustrated that poor psychosocial work environment predicts cardiovascular disease [2,3]. Another limitation is the composition of the sample. A response analysis of the total SCAPIS cohort (*n* = 30,154) [18] illustrated sociodemographic differences between respondents and non-respondents, as response rates differed between persons living in a well-to-do geographical area (participation rate 67%), versus persons living in a poorer geographical area with a large proportion of immigrants (participation rate 37%). Low income and an immigrant background are generally associated with worse risk patterns with respect to CHD. Our study was also limited to those currently working, which could have contributed to healthy worker effects, referring to an inclusion bias towards healthier participants, since ill or injured subjects are not able to work. It is likely that participants with the worst work conditions are on sick-leave, but this is impossible to evaluate with the data available to us. Consequently, our study might be biased towards healthier participants with better work environments, which affects the external validity and may underestimate relationships between psychosocial variables and biomarkers.

## Conclusions

Our findings illustrate the associations between JDC exposures and some biomarkers for cardiovascular disease, and that men and women may differ with respect to psychosocial work exposure and disease susceptibility. The cross-sectional design prevents conclusions on causality, yet the results provide a suggestion of variables for assessment in prospective studies.

## Appendix

**Appendix 1.** Poisson regression for associations between single variables demand and control, and biomarkers for coronary heart disease.

	Hypertension DBP			Hypertension SBP			Low HDL-cholesterol			High LDL-cholesterol			Coronary artery calcification			Metabolic syndrome		
	*Cases*	*PR*	*95% CI*	*Cases*	*PR*	*95% CI*	*Cases*	*PR*	*95% CI*	*Cases*	*PR*	*95% CI*	*Cases*	*PR*	*95% CI*	*Cases*	*PR*	*95% CI*
**Men**																		
Job demand^a^	545	0.93	0.82–1.05	834	0.96	0.88–1.04	377	1.13	0.97–1.33	688	1.02	0.92–1.13	982	1.02	0.95–1.10	612	0.90	0.77–1.04
Job demand^b^	545	0.89	0.78–1.01	834	0.94	0.86–1.03	377	1.08	0.92–1.27	688	1.00	0.90–1.11	982	0.99	0.91–1.07	612	**0.80**	**0.68–0.94**
Job demand^c^	545	0.86	0.77–0.97	834	0.92	0.85–1.00	377	1.04	0.92–1.19	688	0.99	0.89–1.09	982	0.98	0.91–1.06			
Job control^a^	545	0.93	0.81–1.07	834	0.92	0.83–1.01	377	**0.82**	**0.68–0.98**	688	1.04	0.92–1.19	982	0.93	0.86–1.02	612	0.85	0.71–1.01
Job control^b^	545	1.03	0.89–1.20	834	1.00	0.90–1.11	377	0.86	0.71–1.04	688	1.04	0.91–1.19	982	0.95	0.87–1.05	612	0.91	0.75–1.12
Job control^c^	545	1.07	0.93–1.23	834	1.03	0.94–1.13	377	0.89	0.76–1.05	688	1.05	0.92–1.20	982	0.96	0.87–1.05			
**Women**																		
Job demand^a^	455	1.02	0.90–1.16	621	1.05	0.95–1.17	246	1.18	0.98–1.42	692	1.00	0.92–1.10	494	0.95	0.84–1.07	412	0.98	0.73–1.29
Job demand^b^	455	0.98	0.86–1.13	621	1.04	0.93–1.16	246	1.21	0.99–1.48	692	0.99	0.89–1.09	494	0.91	0.80–1.03	412	0.90	0.66–1.23
Job demand^c^	455	0.96	0.85–1.08	621	1.01	0.92–1.12	246	1.15	0.96–1.37	692	0.98	0.89–1.08	494	0.90	0.79–1.02			
Job control^a^	455	**0.71**	**0.61–0.84**	621	**0.81**	**0.71–0.93**	246	**0.67**	**0.54–0.84**	692	0.97	0.85–1.10	494	1.04	0.89–1.22	412	1.19	0.81–1.75
Job control^b^	455	**0.78**	**0.66–0.93**	621	0.90	0.78–1.04	246	0.78	0.61–1.00	692	1.05	0.92–1.20	494	1.16	0.97–1.38	412	1.42	0.92–2.21
Job control^c^	455	**0.84**	**0.72–0.98**	621	0.96	0.84–1.09	246	0.87	0.71–1.07	692	1.08	0.95–1.23	494	**1.18**	**1.00–1.40**			

a Model 1: adjusted for age.

b Model 2: model 1 + higher education, smoking, physical activity, general life stress.

c Model 3: model 2 + BMI.

Bold indicate statistically significant
